# Immunomodulatory Effects of L-Arginine-Modified Silkworm Pupae Protein Enteral Nutrition on Murine Intestinal Morphology and Immunity

**DOI:** 10.3390/ijms26073209

**Published:** 2025-03-30

**Authors:** Rui Yuan, Tianming Wang, Linling Zhang, Lakshmi Jeevithan, Chunxiao Wang, Xiaohui Li, Wenhui Wu

**Affiliations:** 1 Department of Marine Bio-Pharmacology, College of Food Science and Technology, Shanghai Ocean University, Shanghai 201306, China; m220300979@st.shou.edu.cn (R.Y.); m200310993@st.shou.edu.cn (L.Z.); lakshmijeevithan@gmail.com (L.J.);; 2 Department of Biomaterials Engineering, Faculty of Health Sciences, UCAM-Universidad Catolica San-Antonio de Murcia, 30107 Murcia, Spain; 3 National R&D Branch Center for Freshwater Aquatic Products Processing Technology, Shanghai 201306, China

**Keywords:** L-arginine, diabetic enteropathy, immunomodulatory, pupa protein

## Abstract

L-arginine, a semi-essential amino acid, is well-documented for its role in nitric oxide (NO) synthesis, its anti-inflammatory properties, and its modulation of immune responses. Studies suggest it may improve gut barrier function and reduce inflammation in conditions such as colitis or sepsis. However, its specific efficacy in diabetic enteropathy (a complication of diabetes involving intestinal dysfunction, inflammation, and neuropathy) is less studied. To verify whether L-arginine and silkworm pupae components have a role in the treatment of diabetic enteropathy via the regulation of other cytokines and suppression of CD4+ T lymphocyte proliferation, a special medical-purpose formula containing both of these components was tested. For the first time, we have integrated L-arginine and silkworm pupae protein into enteral nutrition formulations for testing its anti-inflammatory potential. We have found that these formulations can improve the characteristics of diabetic intestinal inflammation through nutrient-modulating effects and nutritional efficacy. In addition, L-arginine (L-arginine, L-arg) enhances the immunomodulatory effects of special medical purpose formulas for diabetes mellitus. We utilized an oxidative stress model of small intestinal epithelial cells induced by high glucose and an inflammation model of the small intestine triggered by lipopolysaccharide in mice. The aim was to further investigate the protective effects of L-arginine and enteral nutritional preparations derived from silkworm pupae protein components on the intestinal tract. This research seeks to establish a theoretical foundation for understanding how L-arginine and these nutritional preparations regulate intestinal inflammation in vivo.

## 1. Introduction

Enteral nutrition (EN) preparations consist of substances designed to provide nutritional support via the gastrointestinal tract [1]. They contain nutrients that require minimal chemical digestion or need no digestion [2]. Administering enteral nutrition effectively enhances blood flow to the gastric mucosa and helps maintain the barrier function of the gastrointestinal lining, as well as the health of the intestinal flora, all of which contribute to the recovery of gastrointestinal function [3,4,5]. These preparations are primarily composed of nitrogen sources, carbohydrates, fats, vitamins, minerals, and fiber. With its high nutrient density, multi-targeted functional activity, and good processing characteristics, silkworm pupae protein has been confirmed by many studies as a potential high-quality EN candidate [6,7], especially for metabolic diseases and intestinal inflammation adjuvant therapy [8]. Its mechanism of action covers metabolic regulation [9], immune enhancement, and barrier repair, which is in line with the development trend of “nutrition–function integration” of modern EN preparations.

Diabetes mellitus, a multifactorial metabolic disorder characterized by chronic hyperglycemia and systemic inflammation, underscores the critical interplay between nutrient metabolism and immune dysregulation. Gastrointestinal symptoms are very common in diabetic patients, constituting up to 75% of the total cases [10,11]. Conventional therapeutic strategies often prioritize glycemic control through the pharmacological modulation of insulin signaling or glucose absorption [12,13], yet these approaches frequently overlook the bidirectional interaction between metabolic homeostasis and mucosal immunity [14,15]. Emerging evidence highlights the potential of bioactive enteral nutrition (EN) formulations to synergistically address both metabolic dysfunction and immune imbalance—a paradigm termed “immunometabolic modulation” [16]. Notably, specialized EN formulations enriched with immunonutritional components (e.g., antioxidant peptides, prebiotic fibers, and anti-inflammatory amino acids) may exert dual therapeutic effects.

Immunometabolic modulation refers to how metabolic pathways influence immune cell function and vice versa. Modulation in this context means altering these pathways to either enhance or suppress immune responses, depending on the therapeutic goal. Previous studies suggested that some specific metabolites, such as TCA cycle intermediates, influence the inflammatory activity of immune cells such as T lymphocytes, macrophages, and dendritic cells [17]. There is evidence that bioactive compounds found in foods such as polyphenols, vitamins, fatty acids, and sulforaphane can influence the metabolism of immune cells and their role in managing chronic inflammatory conditions such as obesity and diabetes by modulating metabolic pathways [18]. Overall, in inflammatory diseases, it can help reduce excessive immune responses by altering the metabolism of specific immune cells [19].

Beyond being a building block for protein synthesis, L-arginine promotes intestinal growth and development and maintains the function of the immune barrier of the intestine, including thymotropic effects [20] and the enhancement of the cellular activity of lymphokine-activated killer cells [21]. Based on these effects, L-arginine is important as a special nutrient that can be combined with enteral nutrition preparations to maintain the normal structure and physiological function of the gastrointestinal tract and to prevent intestinal inflammation [22,23].

To assess the potential of our enteral nutrition formulation in alleviating intestinal inflammation, we measured the levels of key cytokines (including IL-6, IL-1β, and TNF-α), antibodies (IgM and IgG), and transporter proteins (PepT1, GLUT2, and FATP4). As a key pro-inflammatory cytokine, elevated levels of IL-6 are often associated with severe intestinal inflammation, as it promotes the recruitment of immune cells and the production of acute-phase proteins [24]. Meanwhile, IL-1β is involved in amplifying the inflammatory response. It contributes to the recruitment of immune cells to the site of inflammation and is linked to the severity of conditions such as inflammatory bowel disease [25]. Studies have shown that TNF-α levels are positively correlated with disease severity, as it amplifies the inflammatory cascade by recruiting immune cells and promoting the release of other pro-inflammatory cytokines [26]. Increased IgG levels have been associated with more severe disease phenotypes in Crohn’s disease. Similarly, IgM levels are often elevated during acute inflammatory responses, indicating active immune engagement [27,28]. PepT1, GLUT2, and FATP4 are associated with gut inflammation, and their expression levels or activity can provide insights into the severity of inflammation [29,30]. sIgA (secretory immunoglobulin A) levels and CD4+ T cells were also tested since these markers provide clearer insights into small intestinal immune responses. In this study, a self-made enteral nutrition preparation was formulated using silkworm pupae protein as the nitrogen source, alongside tapioca starch and corn starch as energy-providing sugars, oligofructose, and isomaltose. Additionally, fish oil and olive oil were included as fat sources to supply calories and essential fatty acids. A variety of vitamins, minerals, and specific nutrients, such as arginine, were also added. L-arginine is a precursor to nitric oxide (NO), a molecule that plays a key role in regulating inflammation and immune function, while nitric oxide can help improve blood flow, support tissue repair, and modulate the inflammatory response [31,32]. Silkworm pupae protein is rich in bioactive peptides and amino acids, which have been shown to possess anti-inflammatory and antioxidant properties [33,34]. This combination of pupae protein and L-arginine was designed to leverage their complementary anti-inflammatory properties; however, for the time being, there are no studies that synergize these two components to test their anti-inflammatory effects. The preparation was tested on a mouse model of small intestinal inflammation induced by LPS, and the results demonstrated that it may possess significant intestinal antibacterial properties by elevating the levels of proteins related to the immune response, including sIgA. Furthermore, the preparation exhibited robust intestinal anti-inflammatory and protective effects.

## 2. Results

### 2.1. Histopathological Manifestations

**Figure 1:**
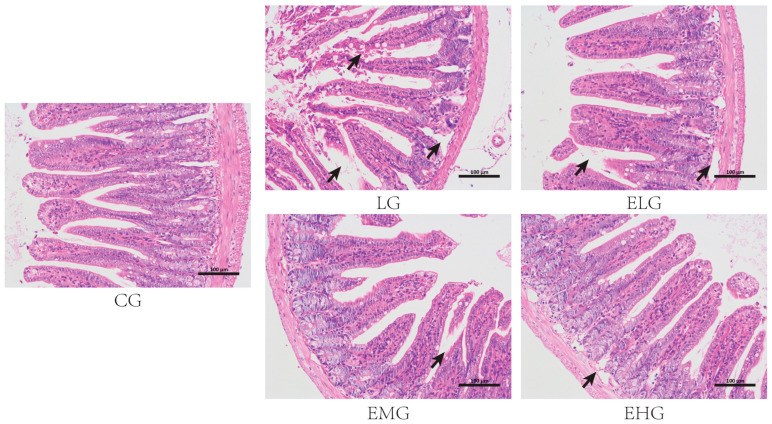
Histopathological manifestations of the intestine in various groups of mice. The control group (CG), negative control group or LPS group (LG), enteral high-dose group (EHG), enteral medium-dose group (EMG), and enteral low-dose group (EHG) are shown. H&E staining showing pathologic changes such as villus shedding and necrosis (black arrow) in different groups. Scale bar: 100 µm.

As shown in Figure 1, the control group (CG, Figure 1) demonstrated intact intestinal architectures with a clearly defined mucosal layer, submucosa, and muscularis propria. Villus structures remained intact without observable damage. The LPS group (LG) exhibited significant pathological alterations, including prominent inflammatory cell infiltration within villus structures, thinning of the muscularis layer, the progressive separation of the muscularis and submucosal layers, and villus detachment and necrotic changes. These findings confirm the successful establishment of intestinal inflammatory injury. The low- and high-dose enteral nutrition groups (ELG/EHG) showed partial improvement characterized by decreased villus detachment and separation between submucosal and muscular layers. The medium-dose enteral nutrition group (EMG) displayed optimal protective effects against inflammatory injury, with relatively higher villus integrity and the distinct stratification of mucosal and submucosal layers.

### 2.2. Blood Parameters

As shown in Table 1, WBC and PLT counts in the LG, ELG, EMG, and EHG groups were significantly lower than those in the CG group (*p* < 0.05). At the same time, they were significantly higher in the LG group than in the ELG, EMG, and EHG groups (*p* < 0.05), with the highest values of WBC and PLT in the EMG group. Compared with the CG group, the RBC values in the LG, ELG, and EHG groups were reduced and produced significant differences (*p* < 0.05), and there was no significant difference from the EMG group. HGB values were significantly higher in the ELG and EMG groups compared to the LG group (*p* < 0.05), and the difference with EHG was not significant.

### 2.3. Cytokine Profiling in Intestinal Tissues

**Figure 2:**
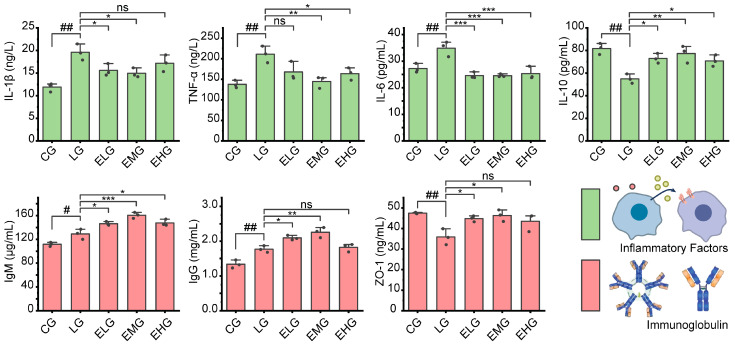
Different intestinal protein levels in all groups of mice. Levels of inflammatory factors, including IL-1β, TNF-α, IL-6, and IL-10, in the small intestinal tissues of mice in all groups and intestinal immunoglobulin levels, including IgM, IgG, and ZO-1, in all groups of mice. For comparison between the CG and the LG: ^#^ *p* < 0.05, ^##^ *p* < 0.01. Comparing groups ELG, EMG, and EHG to group LG: * *p* < 0.05, ** *p* < 0.01, *** *p* < 0.001. ns: not significant (n = 3, one-way ANOVA and Tukey post-test).

As illustrated in Figure 2, significant alterations in inflammatory cytokine levels were observed across experimental groups. Comparing the LPS group (LG) with the control group (CG), pro-inflammatory cytokines including IL-1β, TNF-α, and IL-6 witnessed a significant increase. IL-1β increased from 11.91 ng/L (CG) to 19.60 ng/L (LG) (*p* < 0.001), TNF-α rose from 137.70 ng/L to 210.90 ng/L (*p* < 0.01), and IL-6 was elevated from 27.22 pg/mL to 34.85 pg/mL (*p* < 0.01). For anti-inflammatory cytokines, IL-10 decreased from 81.73 pg/mL (CG) to 54.92 pg/mL (LG) (*p* < 0.01). These findings confirm the presence of pronounced intestinal inflammation in the LG group.

For intervention groups (ELG/EMG/EHG), when compared to the LG group, IL-1β levels in the ELG and EMG exhibited significant reductions to 15.60 ng/L and 14.97 ng/L, respectively (*p* < 0.05), while in the EHG, they showed a non-significant decrease to 17.19 ng/L. For TNF-α levels, the EMG and EHG demonstrated marked declines to 144.70 ng/L (*p* < 0.01) and 163.70 ng/L (*p* < 0.05), respectively, with the EHG showing a further decrease to 167.70 ng/L (non-significant trend). All treatment groups showed robust suppression for IL-6 levels; meanwhile, elevated levels of IL-10 were observed in all intervention groups.

### 2.4. Immunoglobulin and Tight Junction Protein Levels

As demonstrated in Figure 2, for the LG and CG, immunoglobulin levels in intestinal tissue both increased after the injection of the LPS solution: the level of IgM increased from 111.7 µg/mL (CG) to 129.00 µg/mL (LG) (*p* < 0.05) and IgG rose from 1.34 mg/mL to 1.77 mg/mL (*p* < 0.01). ZO-1 decreased from 47.49 ng/mL to 35.89 ng/mL (*p* < 0.01), which may indicate intestinal immune hyperactivation and compromised barrier function in the LG.

Overall, mucosal immunity was enhanced through upregulated immunoglobulin production (IgM/IgG) and restored intestinal barrier integrity via ZO-1 recovery. Medium-dose (EMG) intervention demonstrated superior efficacy, achieving maximal IgM elevation (22.4% vs. LG) and the near-normalization of ZO-1 levels (46.35 ng/mL vs. CG baseline of 47.49 ng/mL).

Of the four cytokines we measured, there was a change in cytokine levels in the direction of no inflammation in small intestinal cells in the diabetic enteropathy model as the EN dose increased from the ELG to the EMG. However, a slight decrease in anti-inflammatory capacity was observed when mice were fed an all-EN diet, meaning that a percentage of EN between 25–100% has the best anti-inflammatory effect. Meanwhile, we will later analyze and discuss the reason why higher EN percentages present in the diet do not lead to better anti-inflammatory effects in the gut.

### 2.5. CD4+ T Lymphocyte Infiltration Analysis

**Figure 3:**
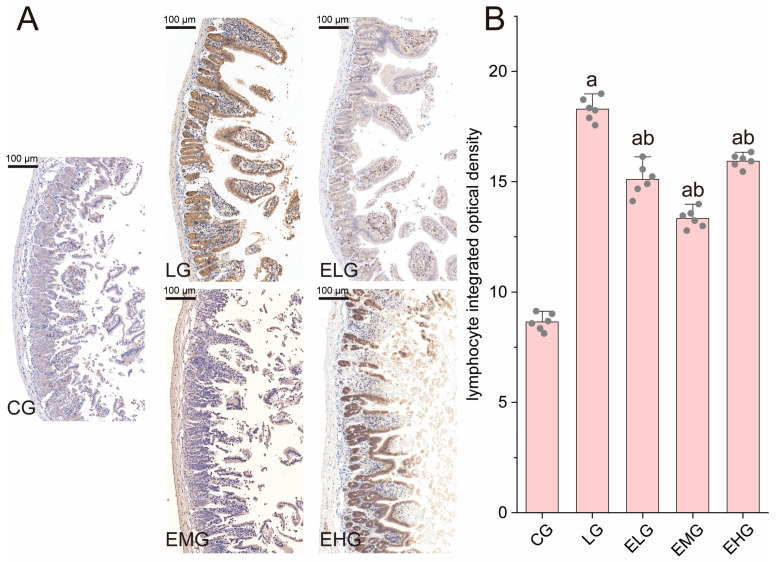
Pathology of intestinal CD4+ T lymphocytes in various groups of mice. (**A**) Immunohistochemistry staining of lymphocyte subsets in the distal ilea tissue of different groups. (**B**) CD4+ T lymphocyte integrated optical density values in the small intestine. Compared with group CG, ^a^ *p* < 0.05; compared with group LG, ^b^ *p* < 0.05 (n = 6, one-way ANOVA and Tukey post-test). Scale bar: 100 µm.

**Figure 4:**
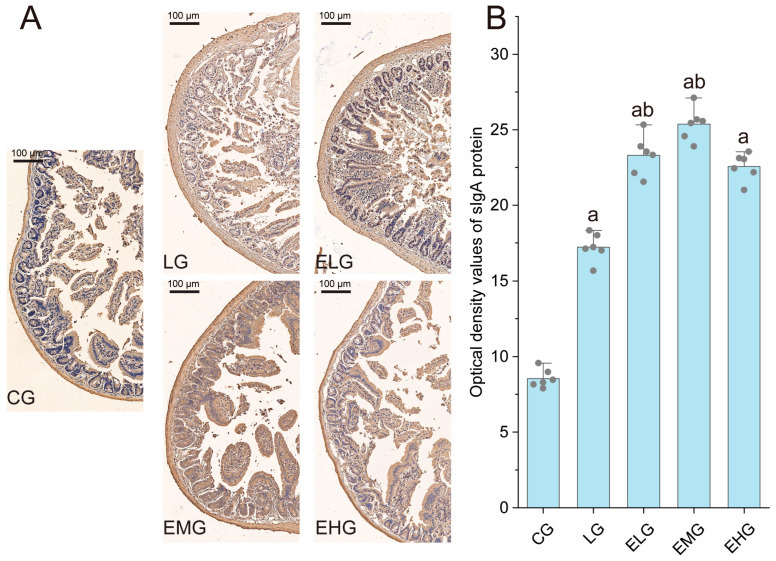
Pathology of sIgA protein in the intestines of various groups of mice. (**A**) Immunohistochemistry staining of sIgA in the distal ilea tissue of different groups. (**B**) Integrated optical density values of sIgA protein in the small intestine of various groups of mice. Compared with group CG, ^a^ *p* < 0.05; compared with group LG, ^b^ *p* < 0.05 (n = 6, one-way ANOVA and Tukey post-test). Scale bar: 100 µm.

As shown in Figure 3A, immunohistochemical staining revealed distinct patterns of CD4+ T lymphocyte distribution across experimental groups. The control group (CG, Figure 3A) exhibited sparse CD4+ T lymphocytes with weak positivity, while the LPS group (LG, Figure 4B) displayed a dense accumulation of brown- or yellow-stained CD4+ T cells in mucosal and submucosal layers, consistent with LPS-induced inflammatory infiltration and barrier dysfunction. In contrast, intervention groups (ELG/EMG/EHG) showed marked reductions in CD4+ T lymphocyte density, with cells localized predominantly in mucosal regions.

Quantitative analysis (Figure 3B) confirmed these observations: Integrated optical density (IOD) values for CD4+ T cells increased significantly from 8.64 ± 0.38 (CG) to 18.29 ± 0.52 in the LG (*p* < 0.05). All treatment groups demonstrated attenuated IOD values compared to the LG. These data may be a sign that the silkworm pupae protein enteral nutrition formulation effectively suppressed CD4+ T lymphocyte proliferation, with the medium-dose group (EMG) exhibiting the most pronounced anti-inflammatory effects. In summary, the weakest immune response was seen in the EMG, which means that the highest immunosuppressive activity may be seen in this group.

### 2.6. Secretory IgA (sIgA) Immunohistochemical Analysis

As illustrated in Figure 4, the control group (CG) exhibited baseline sIgA protein distribution with characteristic brown-yellow staining, while the LPS group (LG) displayed marked sIgA upregulation (*p* < 0.05 versus CG), consistent with LPS-induced mucosal immune hyperactivation. Intervention groups (ELG/EMG/EHG) demonstrated dose-dependent enhancement of sIgA expression, exceeding LG levels.

Quantitative analysis (Figure 4B) confirmed these observations: Integrated optical density (IOD) values for sIgA increased from 8.55 ± 0.62 (CG) to 17.23 ± 0.93 in the LG (*p* < 0.05). All treatment groups exhibited further elevation (*p* < 0.05 versus LG).

## 3. Discussion

Enteral nutrition preparations are a set of substances that can provide nutritional support through the gastrointestinal route by entering some nutrients into the patient’s body that require only chemical digestion or no digestion. When enteral nutrition is given, the blood flow into the gastric mucosa is effectively increased, and the barrier function of the gastrointestinal mucosa, as well as the intestinal flora, can be maintained, which promotes the recovery of the gastrointestinal function. Enteral nutrition preparations are mainly composed of nitrogen sources, sugars, fats, vitamins, minerals, and fiber. Existing L-arginine-containing EN formulations may deal with conditions such as the healing of pressure ulcers [35] or recovery from major gastric surgery [36]. Until now, arginine-rich EN has not been studied for the alleviation of diabetic enteropathy. For the first time, we introduced the anti-inflammatory effect of pupae protein into our EN formulation, compared to other existing EN formulations, and combined pupae with L-arginine in the hope of increasing its anti-inflammatory effect [6].

Silkworm pupae protein is rich in a wide variety of amino acids, with an essential-to-nonessential amino acid ratio that surpasses the amino acid reference pattern proposed by the WHO/FAO [37,38]. Moreover, silkworm pupae protein exhibits significant physiological properties, including the promotion of cell proliferation and antioxidant, anti-inflammatory, and antimicrobial activities [39]. Fish oil, which is abundant in EPA and DHA, has been widely utilized as an immunomodulator [40,41]. Evidence from animal models and clinical trials has demonstrated that long-chain n-3 polyunsaturated fatty acids can reduce inflammatory responses associated with inflammatory bowel disease and enhance intestinal immunity [42]. Olive oil, known for its excellent nutritional and sensory qualities, contains low levels of free fatty acids and has been shown to reduce inflammation risk in the body. Additionally, arginine plays a critical role in enhancing immune function, supporting growth and development, protecting intestinal mucosa, and promoting metabolism, making it an essential component in clinical nutritional therapy [43,44].

CD4+ T lymphocytes and CD8+ T lymphocytes are present in the mucosa of the gastrointestinal tract of the organism and reflect the immunity of the organism. CD4+ T lymphocytes are the main effector cells when the organism suffers from enteritis [45]. In this study, we found that LPS led to the proliferation of large numbers of CD4+ T lymphocytes, and the feeding of mice with enteral nutritional preparations reduced the proliferation of large numbers of CD4+ T lymphocytes and maintained the stability of the microenvironment of the small intestine.

Diarrhea is a typical symptom of intestinal inflammation, and we found that the enteral nutrition preparation group was possibly able to slow down LPS-induced diarrhea and weight loss. Hematological analysis demonstrated that intraperitoneal LPS administration induced a significant reduction in key blood biomarkers. Meanwhile, feeding the enteral nutrition preparation with silkworm pupae protein components elevated WBC, RBC, HGB, and PLT levels in mice.

Intestinal sIgA, together with other immune factors in the intestine, participates in immunosurveillance, immunostabilization, and immunoregulation through multiple pathways to ensure the normal physiological function of the intestine [46,47,48]. sIgA is an important antibody protein in the development and regression of neonatal gastroenteropathies, stress ulcers [49], and other diseases. In this study, we found that LPS led to an increase in the expression of a large number of sIgA proteins. sIgA could be further elevated and intestinal immunity could be improved after mice were fed with enteral nutrition preparations.

These findings may align with several immunometabolic pathways. L-arginine is known to modulate the mechanistic target of the rapamycin (mTOR) signaling pathway, which plays a critical role in T cell metabolism and function. In CD4+ T cells, mTOR signaling influences differentiation into effector or regulatory T cells (Tregs). L-arginine enhances mTORC1 activity, promoting the differentiation of Th1 and Th17 cells, which are pro-inflammatory. However, at optimal levels, it can also support Treg differentiation, contributing to immune homeostasis [50,51]. Meanwhile, it also acts as a substrate for nitric oxide (NO) production, which can indirectly influence mTOR signaling by modulating cellular redox states and energy metabolism [51].

It is noteworthy that the EMG was more tolerant to LPS-induced inflammation than the EHG in terms of most of the results (including levels of inflammatory factors and immunoglobulins, as well as immunohistochemical staining). This suggests that there is room for improvement in the nutritional comprehensiveness or nutritional dosage of our EN. L-arginine can be both beneficial and detrimental, depending on context and dosage, which has previously been reported as the “arginine paradox” [52]. Excessive arginine may shift its metabolism (for instance, toward nitric oxide or polyamine pathways) in ways that do not necessarily favor anti-inflammation in the intestine [53]. Moreover, another reason why the EMG outperformed the EHG is that eating a more typical chow (even partially) can preserve normal chewing, gastric emptying, and gut motility patterns, which may help reduce local inflammation by maintaining healthy mechanical and secretory functions in the gastrointestinal tract.

In conclusion, although our LPS-induced intestinal inflammation model may not fully replicate the reality of diabetic enteropathy in humans, as the microbiota data are not yet being fully explored, which limits the mechanistic conclusions related to microenvironments, this study is nonetheless instructive. The L-arginine-rich silkworm pupae protein component in our enteral nutrition demonstrates excellent nutritional benefits. It effectively alleviates LPS-induced inflammation in the small intestine of mice, reduces tissue damage, restores intestinal immune function, and preserves microenvironmental homeostasis. These findings offer valuable insights for preventing and treating intestinal inflammation, improving intestinal health, and providing a scientific basis for developing specialized medical food formulations.

## 4. Materials and Methods

### 4.1. Silkworm Pupae Protein-Based Enteral Nutrition Preparations

The enteral nutrition preparations used in this study were formulated with the ingredients described in Table 2. The nutrient composition and ratios remained largely consistent with previous studies [54,55]. The preparation process involved thoroughly mixing the components to achieve a homogenous blend. The resulting mixture was stored at −20 °C shortly (less than 1 day) until further use. We prepare formulations daily according to the amount of food consumption of the mice, so that almost all of the preparations to which the mice are exposed are freshly formulated within 24 h.

### 4.2. Mice Treatments

All mouse experiments were conducted in accordance with the guidelines of the Committee on the Care and Use of Laboratory Animals after their approval. To induce gut inflammation, the mice were injected with LPS intraperitoneally [56,57] with a single dose of 5 mg/kg, which is consistent with previous reports given that LPS can induce inflammation in different tissues including the gut [58,59,60]. Before the experiment, mice had free access to food and fresh water and were kept at 22–25 °C with a humidity of 30–40% and a 12 h/12 h light/dark cycle.

Four-week-old female C57BL/6 mice (12–17 g) obtained from SPF biotech Ltd. (Beijing, China) were randomly divided into 5 groups of 10 mice each: the control group (CG), the negative control group or LPS group (LG), the enteral high-dose group (EHG), the enteral medium-dose group (EMG), and the enteral low-dose group (EHG). There were no significant differences in the body weight of mice between groups. In both the CG and LG, mice were fed with normal treats (SPF-F02-002, SPF biotech) for 2 weeks and then injected with sterile saline (CG) or LPS solution (LG). After feeding with the enteral nutritional preparation (EHG), 50% enteral nutrition and 50% normal chow (EMG), or 25% enteral nutrition and 75% normal chow (ELG) for 2 weeks, three groups of mice were injected with the LPS solution described above.

Throughout the experimental procedure, no significant preference was observed for the consumption of enteral nutrition preparations versus normal chow in the diets of the above groups of mice. On average, the daily food intake of mice in each group was 3.7 g (including normal chow and enteral nutrition), and the difference in the food intake of mice between groups was insignificant.

### 4.3. Tissue Collection and Histological Analyses

After 4 h of injecting LPS solution or sterile saline, all mice were sacrificed for sampling [61,62]. For histological analyses, an approximately 3 cm segment of the distal ilea was excised and transferred in buffered 10% formaldehyde to ensure complete fixation. After fixation at 4 °C for 12 h, paraffin embedding was carried out and the sample was cut into 3 to 5 μm sections. Sections were stained with H&E staining [63] and other histologic analysis methods, and experimental methods remained consistent with those previously reported or were mildly modified as appropriate.

### 4.4. Immunohistochemistry

For immunohistochemistry, 3 to 5 μm sections were used with the following monoclonal antibodies: anti-CD4 (ServiceBio, Wuhan, China) and anti-sIgA (ServiceBio, Wuhan, China). Most of the previously reported protocols were used [64].

Tissue sections were deparaffinized in xylene and rehydrated through a graded ethanol series (100%, 95%, 80%, and 70%) followed by distilled water. Heat-induced antigen retrieval was performed in citrate buffer (pH 6.0) using microwave irradiation. Sections were heated at medium power for 10 min, incubated without heating for an additional 10 min, and then treated at medium-low power for 7 min. After cooling to room temperature, slides were washed three times (5 min each) in PBS with gentle shaking. Non-specific binding sites were blocked with an appropriate serum for 1 h at room temperature. A diaminobenzidine (DAB) substrate was applied to visualize immunoreactivity, and the reaction was monitored microscopically until the optimal signal-to-background contrast was achieved.

### 4.5. Quantification of Labeled Cells

Representative fields of CD4 and sIgA staining were photographed with a digital camera. The number of positive cells was analyzed by integrating optical density values using Image J Fiji distribution [65,66]. Results are presented as the relative optical density.

### 4.6. Blood Parameter Analysis

Blood samples were collected after 4 h of injecting LPS solution or sterile saline. Retro-orbital bleeding under anesthesia was adopted. Approximately 0.5–1.0 mL of blood was obtained from each mouse using a syringe. The collected blood samples were immediately transferred into EDTA-coated tubes to prevent coagulation. The samples were then gently mixed and stored on ice.

Hematological parameters, including white blood cell (WBC) count, red blood cell (RBC) count, blood platelet (PLT) count, and hemoglobin (HGB) concentration, were measured using an automated blood cell analyzer (BC-2800Vet, Mindray Animal, Shenzhen, China).

### 4.7. Quantification of Inflammatory Cytokines and Immune Mediators Using ELISA

The small intestinal tissue samples described above were retrieved from storage at −80 °C and allowed to equilibrate at 4 °C for 10 min. Approximately 1 g of tissue was weighed and homogenized in 9 mL of ice-cold phosphate-buffered saline (PBS, pH 7.4) using a mechanical tissue homogenizer, with all procedures performed under ice-cold conditions to minimize protein degradation. The homogenate was centrifuged at 4 °C for 20 min at 1000× *g*. Following centrifugation, the supernatant was carefully collected for immediate analysis, while residual aliquots were snap-frozen in liquid nitrogen and stored at −80 °C for subsequent assays. IL-6 (Cat# E-EL-M0044c), IL-1β (Cat# E-EL-M0037c), TNF-α (Cat# E-EL-M0049c), IgM (Cat# E-EL-M0695c), IgG (Cat# E-EL-M0689c), PepT1 (Cat# E-EL-M0566c), GLUT2 (Cat# E-EL-M0562c), and FATP4 (Cat# SEG555Ra) ELISA kits from Winter Song Boye Biotechnology (Beijing, China) were used according to the manufacturer’s instructions.

### 4.8. Statistical Analysis

All data obtained from assays were compared using analysis of variance (ANOVA), followed by a Tukey multiple comparisons post-test, with *p* < 0.05 indicating that the data were different and statistically significant. GraphPad Prism (Version 9.0) and a self-made python script that imports the matplotlib and scipy libraries were used for data analysis [67,68].

## Figures and Tables

**Table 1:** Blood indices of mice in different groups. Parameters including white blood cell (WBC) count, red blood cell (RBC) count, hemoglobin (HGB) concentration, and blood platelet (PLT) count are presented below. After ANOVA analysis, multiple comparison correction methods including Benferroni were adopted. Compared with group CG, ^a^ *p* < 0.05; compared with group LG, ^b^ *p* < 0.05 (n = 3, one-way ANOVA and Tukey post-test).

Indicators	CG	LG	ELG	EMG	EHG
WBC (10^9^/L)	2 ± 0.1	1.1 ± 0.1 ^a^	1.57 ± 0.21 ^ab^	1.67 ± 0.06 ^ab^	1.5 ± 0.1 ^ab^
RBC (10^12^/L)	11.5 ± 0.41	8.42 ± 0.34 ^a^	10.19 ± 0.13 ^ab^	10.54 ± 0.37 ^b^	9.78 ± 0.48 ^ab^
HGB (g/L)	173.33 ± 2.89	138.67 ± 3.21 ^a^	153 ± 2 ^ab^	165 ± 5 ^b^	146.67 ± 4.73 ^a^
PLT (10^9^/L)	514.33 ± 11.68	229.67 ± 14.74 ^a^	280 ± 7.55 ^ab^	293 ± 6.56 ^ab^	273.33 ± 17.56 ^ab^

**Table 2:** Ingredients of silkworm pupae protein-based enteral nutrition preparations.

Ingredients	Grams per 100 g
Silkworm pupae protein	23.00
Fish oil	5.00
Olive oil	15.00
Corn starch	12.00
Tapioca starch	17.50
Fructooligosaccharides	9.00
Isomaltulose	5.00
L-arginine	0.25
Vitamins and minerals	3.25
Dietary fiber (inulin)	10.00

## Data Availability

The data underlying this study are available within the manuscript.
